# Stepwise Palatal Prosthetic Rehabilitation After Pediatric Ischemic Stroke

**DOI:** 10.3390/reports9010078

**Published:** 2026-03-06

**Authors:** Satoru Kusaka, Yuria Asao, Tatsuya Akitomo, Yuko Iwamoto, Ryota Nomura

**Affiliations:** 1 Department of Pediatric Dentistry, Hiroshima University Hospital, Hiroshima 734-8551, Japan; 2 Department of Pediatric Dentistry, Graduate School of Biomedical and Health Sciences, Hiroshima University, Hiroshima 734-8553, Japan; yuriaasao@hiroshima-u.ac.jp (Y.A.); takitomo@hiroshima-u.ac.jp (T.A.); yuko-tulip@hiroshima-u.ac.jp (Y.I.); rnomura@hiroshima-u.ac.jp (R.N.)

**Keywords:** pediatric ischemic stroke, dysphagia, palatal augmentation prosthesis, palatal lift prosthesis, oral motor rehabilitation, velopharyngeal dysfunction, oral appliance

## Abstract

Pediatric ischemic stroke is rare but may result in severe oral dysfunction. Evidence for prosthetic oral rehabilitation is well established in adults, whereas pediatric data remains limited. We report a pediatric patient with persistent dysphagia and articulatory impairment following recurrent ischemic stroke who underwent stepwise palatal prosthetic intervention. Treatment began with a palatal augmentation prosthesis to establish tolerance and promote tongue–palate contact, followed by a palatal lift prosthesis providing gentle velopharyngeal support. Tongue pressure measurements, oral diadochokinesis, and speech intelligibility improved during appliance use, with gains largely maintained after discontinuation, suggesting motor relearning rather than transient mechanical assistance. This case illustrates the potential value of a tolerance-oriented, stepwise prosthetic strategy in pediatric stroke rehabilitation and underscores the need for individualized adjustment and cautious interpretation of functional metrics.

**Figure 1:**
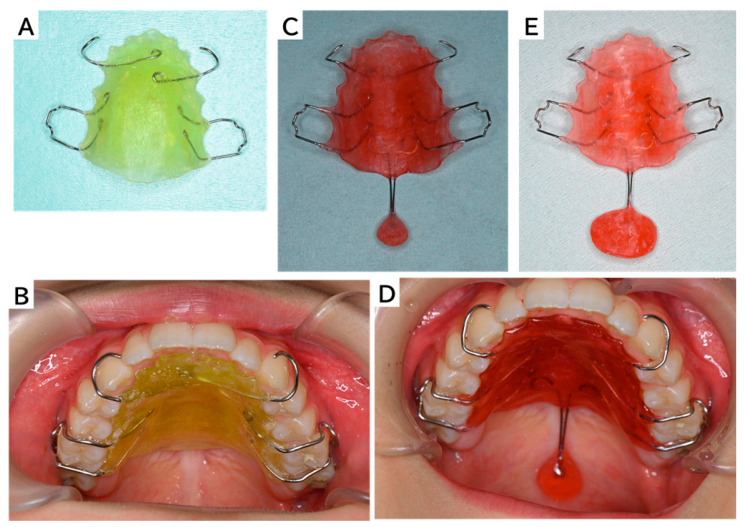
Three types of intraoral appliances for improving oral function. A female patient aged 13 years and 3 months experienced an ischemic stroke followed by a recurrence 3 months later, resulting in limited mouth opening, dysphagia, and severe articulation difficulties. At the age of 13 years and 4 months, she was referred to our department and was supported by continuous oral hygiene management and functional training throughout hospitalization and rehabilitation. One year and 10 months after the first stroke, significant oral dysfunction persisted. Unlike conditions involving structural defects, the present case primarily reflected neuromotor impairment. Consequently, immediate definitive prosthetic correction was not considered appropriate. A graded, tolerance-oriented strategy was selected to facilitate adaptation to intraoral stimulation and to support motor relearning, particularly given the sensory sensitivity often observed in pediatric patients. A stepwise intraoral prosthetic intervention was therefore started to enhance tongue–palate contact and velopharyngeal closure. At the age of 15 years and 1 month, a palatal augmentation prosthesis (PAP) was fitted [1] (**A**,**B**). The basic shape of the PAP was designed to promote contact between the tongue and palate, aiming to reduce resistance to the intraoral prosthesis, lessen discomfort, and encourage habitual daily use. She was able to perform her daily activities while wearing the device. After wearing the device for 1 month, the patient had established the habit of wearing the prosthesis and had adapted to the discomfort associated with wearing it. She then transitioned to a second oral appliance, a PAP with a movable palatal lift (PLP) [2], with the aim of familiarizing the patient with soft palate contact and introducing mild velopharyngeal support (**C**,**D**). A small movable palatal lift attached to the PAP using superelastic Ni-Ti wire enabled gentle contact with the soft palate or its mild elevation [3,4]. We aimed to provide proprioceptive stimulation without restricting tongue-based movement. Despite the device constantly contacting her soft palate, she did not experience any gag reflexes. She also continued swallowing training with the device in place for 5 months, transitioning to the next device at age 15 years and 10 months. The third device was a modification of the second prosthesis to compensate for asymmetry in the nasopharyngeal closure (**E**). It was primarily designed to expand the lift elements on the right side while adjusting the wire angle to generate an active lift force. For 11 months, the patient wore the device while living normally and continued swallowing exercises at regular monthly hospital visits. The swallowing exercises enabled the patient to swallow regular food without using the device. At 16 years and 9 months of age, use of the device and active swallowing exercises were discontinued.

**Figure 2:**
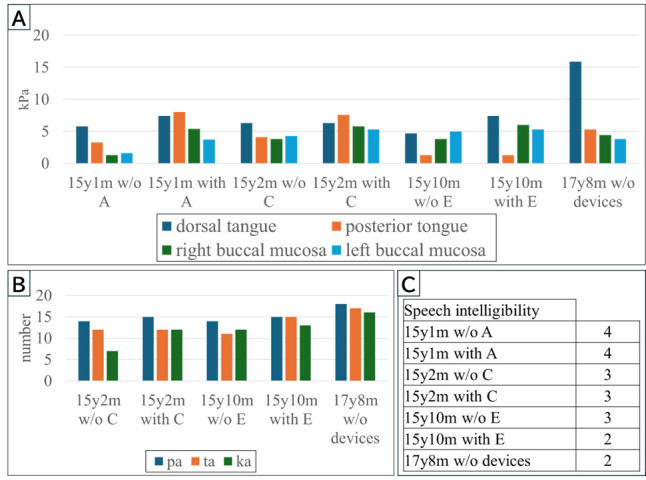
Oral function evaluation using objective devices. Three types of examinations were conducted continuously over time to evaluate improvements in oral function resulting from the use of oral appliances. Tongue pressure was measured using the JMS TPM-02E device. The probe was inserted between the dorsal tongue, posterior tongue, and left and right buccal mucosa to measure muscle pressure at the following sites: tongue–hard palate, tongue–soft palate, and buccal mucosa—dental arch. Oral diadochokinesis (ODK; “pa–ta–ka”) was assessed using the Kuchiken App, and the patient was instructed to produce the sounds “pa,” “ta,” and “ka” as many times as possible for 10 s each, with the score recorded by the app. Speech intelligibility was rated subjectively by the examining dentist using a five-point scale: 1 = easily understood; 2 = occasional unintelligible words; 3 = understood only when the listener knows the context; 4 = occasional intelligible words; 5 = unintelligible. For each examination, the pre-treatment intervention score was used as the baseline to evaluate the results of the treatment intervention. Use of a palatal augmentation prosthesis (PAP) and a PAP with a movable palatal lift (PAP–PLP) increased anterior tongue and bilateral cheek pressure (**A**). Tongue pressure values represent absolute measurements (kPa) obtained using the JMS TPM-02E device. Elevated posterior tongue pressure during use of the second appliance resulted from mechanical contact with the movable lift. After discontinuation, anterior tongue pressure remained above baseline, suggesting that motor relearning had occurred rather than dependence on the device. ODK repetition counts (“pa–ta–ka”) improved during use of each appliance (**B**). ODK performance is expressed as repetition counts per 10 s. Speech intelligibility improved steadily from a score of 4 at baseline to 2 at the end of treatment. Improvements persisted after appliance removal, demonstrating lasting neuromotor adaptation. Speech intelligibility scores also improved over time, reaching a score of 2 with the third appliance (**C**). Additionally, during follow-up, the score remained at 2 even without appliance use. Residual dysphagia and articulation disorders may occur after stroke, and intraoral prostheses have been reported as effective rehabilitation aids in adults [1,2,3,4]. However, because pediatric stroke is rare, reports of oral appliance therapy in children are extremely limited. In this case, stepwise use of intraoral prostheses led to functional improvement in a pediatric patient. Speech intelligibility ratings were based on a single examiner’s perceptual evaluation, which may introduce observer-dependent bias. Future studies incorporating multiple evaluators or instrumental acoustic analysis would strengthen objectivity. Tongue pressure measurements should also be interpreted cautiously [5]. Because the measurement probe has a finite size, recorded values may be influenced not only by muscular force generation but also by spatial constraints and tongue displacement mechanics. In cases where tongue mobility is limited, insertion of a relatively large probe may reduce movement amplitude and potentially produce higher apparent pressure values independent of true strength changes.

Timeline of intervention: PAP (1 month) → PAP with movable PLP (5 months) → modified asymmetric PLP (11 months).

The case highlights that a gradual, tolerance-oriented introduction of prostheses may be a useful rehabilitation strategy in pediatric strokes, while also underscoring the need for individualized device design and further clinical research. Because this is a single-patient observation, generalization should be made cautiously. In the present case, the patient did not exhibit severe feeding intolerance or nutritional instability, allowing safe implementation of gradual prosthetic intervention. The stepwise protocol may therefore be most applicable to patients with stable general health, sufficient cognitive understanding, and adequate tolerance to intraoral stimulation. Conversely, careful consideration may be required in patients presenting with marked oral hypersensitivity, pronounced gag reflex, significant nutritional compromise, or limited ability to cooperate with device use and training. Individual patient characteristics, including medical status and behavioral adaptability, should guide prosthetic decision-making.

Because this report reflects the currently available observation period, evaluation of longer-term outcomes remains a subject for future study.

## Data Availability

The original data presented in the study are included in the article, further inquiries can be directed to the corresponding author.
